# School- and intervention-related factors associated with institutionalization of health promotion interventions in elementary schools

**DOI:** 10.24095/hpcdp.44.4.03

**Published:** 2024-04

**Authors:** Robert J. Wellman, Erin K. O’Loughlin, Katerina Maximova, Jodi Kalubi, Teodora Riglea, Jennifer O’Loughlin

**Affiliations:** 1 Department of Population and Quantitative Health Sciences, Division of Preventive and Behavioral Medicine, UMass Chan Medical School, Worcester, Massachusetts, United States; 2 Centre de recherche du Centre hospitalier de l’Universit de Montral (CRCHUM), Montral, Quebec, Canada; 3 Faculty of Kinesiology and Physical Education, University of Toronto, Toronto, Ontario, Canada; 4 MAP Centre for Urban Health Solutions, Li Ka Shing Knowledge Institute, St. Michael’s Hospital, Toronto, Ontario, Canada; 5 Dalla Lana School of Public Health, University of Toronto, Toronto, Ontario, Canada; 6 Department of Social and Preventive Medicine, Universit de Montral, Montral, Quebec, Canada; 7 Centre de recherche en sant publique (CReSP), Universit de Montral, and CIUSSS du Centre-Sud-de-l’le-de-Montral, Montral, Quebec, Canada

**Keywords:** health-promoting schools, interventions, cross-sectional, sustainability, institutionalization

## Abstract

**Introduction:**

Long-term availability of health-promoting interventions (HPIs) in school settings can translate into health benefits for children. However, little is known about factors associated with HPI institutionalization in schools. In this study, we identified correlates of the institutionalization of HPIs offered in elementary schools in Quebec, Canada.

**Methods:**

In two-part, structured telephone interviews over three academic years (2016–2019), elementary school principals (or their designees) throughout Quebec identified an index HPI offered at least once in their school during the previous three years, and were asked whether it was institutionalized (i.e. explicitly written in the school’s educational project, e.g. in the form of educational objectives and means of achieving them). We examined associations between institutionalization and 10 school-related and 16HPI-related characteristics in univariable and multivariable logistic regression analyses.

**Results:**

School key informants (n=163) reported on 147 different HPIs that had been available in their schools in the past three years, 56% of which were institutionalized. Three aspects of school culture—parent/community engagement with the school, school/teacher commitment to student health and school physical environment—were positively associated with HPI institutionalization. HPI-related characteristics positively associated with HPI institutionalization included number of competencies addressed by the HPI, number of teaching strategies employed, modifications made to the HPI prior to or during implementation and perceived success of the HPI. Inviting families or community groups to participate in the HPI was inversely associated with institutionalization.

**Conclusion:**

Better understanding of factors associated with HPI institutionalization may inform the development of school-based HPIs that have the potential for sustainability.

**Highlights**

- Parent/community engagement with the school, school/teacher commitment to student health and school physical environment were positively related to health-promoting intervention (HPI) institutionalization.
- HPIs that included more competencies, that employed more teaching strategies, that were modified prior to or during implementation and that were seen as more successful were more likely to be institutionalized.
- Perceived success was unrelated to formal evaluation of HPIs.
- Understanding school- and HPIrelated factors associated with HPI institutionalization may help optimize sustainability.
- We suggest incentivizing evaluation of HPI effectiveness to guard against ending effective or sustaining ineffective interventions.

## Introduction

Health-promoting interventions (HPIs) targeting children and youth support the development of a wide range of positive health behaviours. Schools are ideal settings for both long-term and continuous HPI delivery because all youth attend school early in life when health-related attitudes and behaviours are shaped.^1^,^2^ Indeed, in order to accrue long-term benefits, a core feature of HPIs that requires consideration, in addition to their effectiveness, is their sustainability (i.e. continuation and durability).^3^

Little is known about how to sustain HPIs, although accumulating evidence suggests that sustainability is challenging, especially in complex settings such as schools. Follow-up of two highly resourced school-based programs (i.e. the Child and Adolescent Trial for Cardiovascular Health [CATCH] program in the US^4^ and the KidsMatter mental health promotion program in Australia^5^) indicates that most schools did not continue to deliver the program in full or at the same intensity after the first year of implementation.^4^,^6^ In alignment with these findings, a recent systematic review on school-based HPI sustainability in high-income countries indicated that none of the 18 programs studied were sustained in their entirety after funding had ended, when evaluation of sustainability occurred one to five years after the intervention.^7^

A key indicator of HPI sustainability is institutionalization, which refers to the formal integration of health promotion activities into the established structures and operations of a school.^8^ Specifically, policies, programs and systems are created or adapted within schools to support and sustain the HPI over the long term. Institutionalization not only optimizes HPI implementation over time but also fosters a school culture that is supportive of health and well-being.^9^ Institutionalization may be a critical component in achieving long-term HPI success and should be prioritized in future research and practice.

Despite the importance of institutionalization for long-term success, the evidence on factors associated with institutionalization of school-based HPIs is nascent. Indeed, a systematic review of 24 studies on sustainability of HPIs found that most focussed solely on early implementation, with only two specifically examining institutionalization.^7^ The few existing qualitative studies identify barriers to institutionalization, including lack of resources, lack of teacher and parent “buy in” and involvement, changes in school leadership, staffing, culture and student needs, lack of staff training, incompatibility of the HPI with the school environment, goals, mandates and, finally, inadequate “know-how” to implement and evaluate the HPI.^6^,^8^,^10^

In the current study, we defined HPIs as activities complementary to the educational curriculum that are offered to all students during class time at no cost, and for which student attendance is expected. We operationalized institutionalization as written incorporation of the HPI into the school’s educational project (*projet ducatif*), which details the school’s values, policy orientations and educational objectives, along with tangible actions, indicators and evaluation measures to ensure that the educational project is achieved.^11^

Periodically (typically, every five years), the Quebec Ministry of Education updates its strategic educational plan, which “defines... the main orientations to be adopted by the education system and specifies the expected results.”^11^^, p.6^ School boards and the schools they oversee then design their educational projects in alignment with the Ministry’s strategic plan, and schools report progress on the educational project to their respective school boards and the public annually. 

We do not consider institutionalization equivalent to sustainability of the HPI, which is a broader construct. We identified correlates of HPI institutionalization from an array of school- and HPI-related characteristics. In addition, we studied a wide range of different types of HPIs addressing a multitude of health issues in a large sample of elementary schools. To select potential correlates, we drew on diffusion theory,^12^ which describes four phases of HPI delivery, including planning, implementation, sustainability and scale-up. Importantly, our adapted conceptual model^13^ also draws on socioecological theory^14^ to situate HPI delivery within both the school context and the broader contexts of the community and the educational and political systems. Finally, we focussed on elementary schools because their context, resources and student needs differ markedly from high schools.

## Methods

Project Prome*SS*^15^ is designed to investigate social inequalities in HPI availability in elementary and high schools across Quebec, Canada, using cross-sectional surveys. In the years 2016 through 2019, data were collected from school principals, vice-principals or teachers in a convenience sample of 171 public primary schools in the province. The details have been described elsewhere.^13^

***Ethics approval***

Prome*SS* was approved by the Centre hospitalier de l’Universit de Montral (CHUM) Ethics Review Board. The CHUM certificate of ethics approval (2013-4130, CE 12.307) was submitted to all eligible school boards and principals upon request. School boards provided consent to approach the schools within their jurisdiction, and each school principal provided their consent to participate.

***Procedures***

Data collection procedures are detailed elsewhere.^13^,^16^ Briefly, data were collected in a two-part, structured telephone interview (median length = 52 min) administered by trained interviewers in French or English. School principals were solicited; if they had not worked in their current school at least six months or were unavailable, they were asked to nominate another key informant (i.e. a vice-principal or other staff member). In the first part of the interview, school key informants provided information on characteristics of the school, school key informant (i.e. position, years working in the school) and availability of HPIs. 

In the second part of the interview, participants responded to the following instruction: “The following questions pertain to ONE specific health-promoting intervention that is currently being offered in your school or that was offered within the last three years. If your school is currently offering a tobacco control intervention or has offered one in the last three years, please answer the following questions with reference to this tobacco control intervention. If your school does not currently offer a tobacco control intervention or has not offered one in the last three years, then think of any health-promoting intervention that is current or that was offered in the last three years. Please answer the following questions with that one intervention in mind. Note that the response choices are in the past tense although we understand that the intervention may be ongoing.” The Prome*SS* I 2017-2019 elementary school questionnaires (Adoption of HPIs [part 1] and Implementation of HPIs [part 2]) are available here: https://www.celphie.ca/promess-questionnaires. 

If no HPIs were offered in the school within the preceding three years, the questions about an index HPI were skipped. After an index HPI was selected, participants responded to in-depth questions on the health issue addressed and the selection, planning, implementation and institutionalization of the index HPI. Prome*SS* questionnaire items were developed de novo or drawn or adapted from previous studies.^17^

***Study variables***

Institutionalization of the index HPI was measured by asking: “Is the intervention explicitly written in your school’s orientation plan (e.g. the educational project, the success plan or others)?” Response options were “no” or “yes.” 

We assessed 10 school-related characteristics. Six referred to school structure or student demographic characteristics: (1)school deprivation level; (2) size of population centre served by the school; (3) language of instruction (French or English, determined by the school board); (4) number of students in school; (5) teacher turnover; and (6) principal turnover. Four referred to health-promoting school culture: (7) parent/community engagement in school; (8) school/teacher commitment to student health; (9) school physical environment; and (10) ease of principal leadership (i.e. how easy or difficult it is for the principal to accomplish seven tasks; Table 1). 

**Table 1:** Questionnaire items, response options and recoding of response options for analysis—school-related characteristics

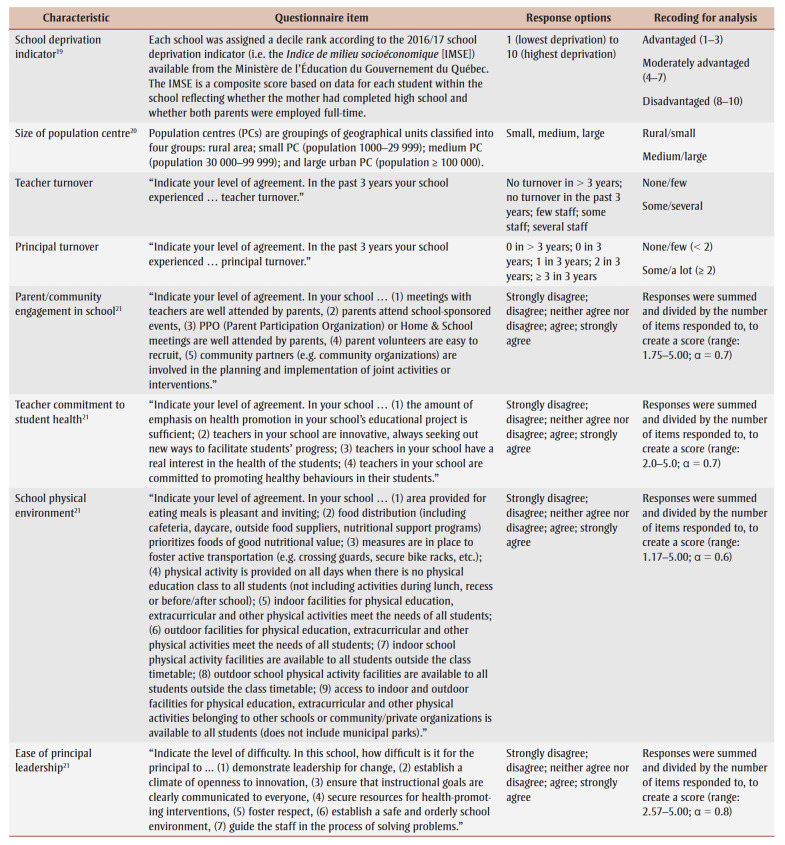

“Health-promoting school culture” encompasses the school contextual elements (e.g. values, expectations, resources) that influence HPI implementation.^18^ It is drawn from the World Health Organization’s Health Promoting Schools framework, which focusses on (1) incorporating HPIs into the school’s formal curriculum; (2)promoting student health and well-being by promoting certain values and attitudes and providing a favourable physical environment; and (3) engaging with students’ families and communities.^18^

The scales measuring parent/community engagement, school/teacher commitment and physical environment were developed through exploratory factor analysis. The scale measuring ease of principal leadership was developed de novo.^18^ Table 1 presents the derivation, wording and coding of school-related characteristics.

Sixteen characteristics of the index HPI potentially related to institutionalization included: (1) number of years HPI offered in school; (2) whole school approach to HPI (i.e. all grades received HPI); (3) HPI designer; (4) number of core competencies addressed by the HPI^22^; (5) number of teaching strategies employed; (6) program champion present; (7) nature of HPI animators (i.e. the individuals who deliver the HPI; see list of examples in Table 2); (8) families invited to participate in HPI; (9) community groups invited to participate in HPI; (10) who was responsible for implementing HPI?; (11) school board involved in HPI implementation; (12)number of complementary initiatives in school during HPI implementation; (13) modifications made to HPI; (14) perceived success of HPI; (15)HPI produced changes; and (16) evaluation effort. Table 2 details questionnaire items, response options and coding for analyses of the HPI-related characteristics.

**Table 2:** Questionnaire items, response options and recoding of response options for analysis—HPI-related characteristics

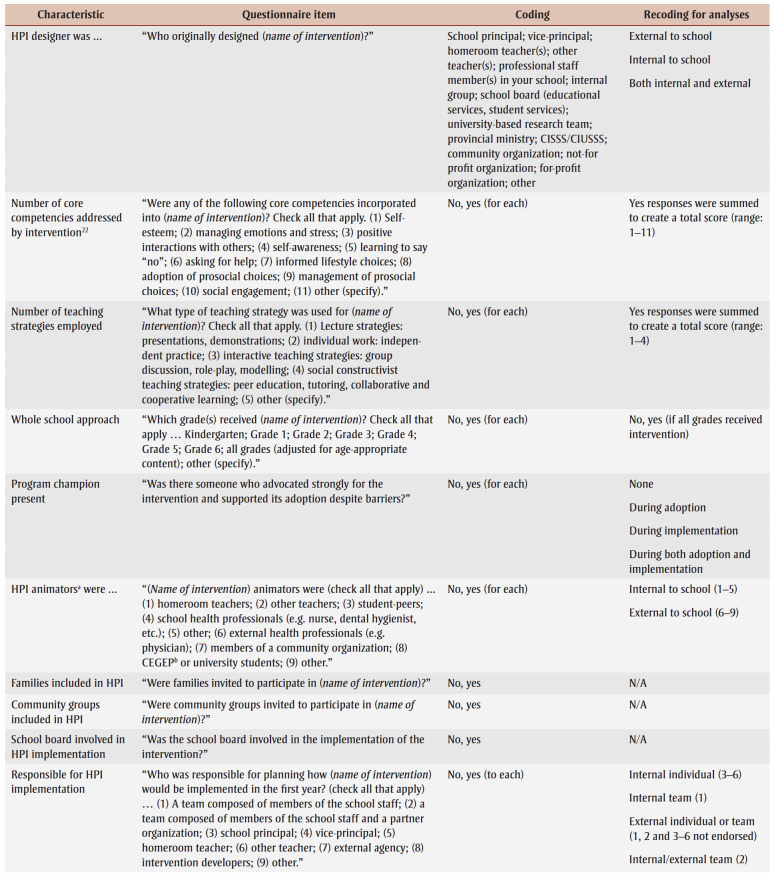 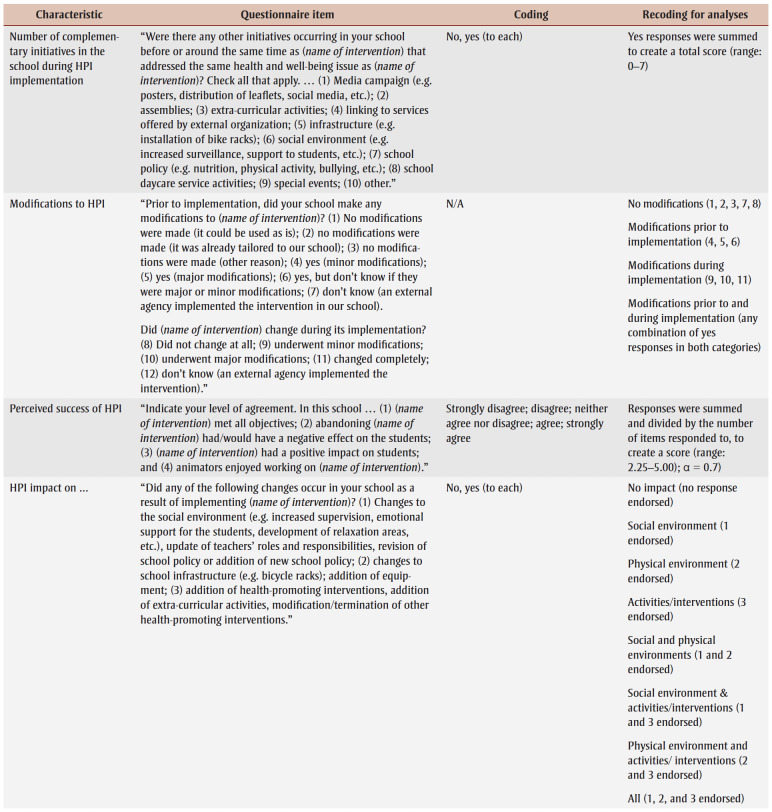 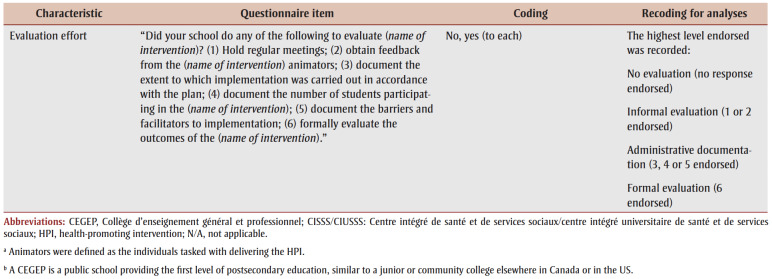

***Data analysis***

After computing descriptive statistics, we estimated associations for each potential correlate in two logistic regression models—an unadjusted model and a model adjusted for school deprivation level, population centre size, language of instruction and number of students. We did not estimate a model containing all potential correlates, as such models may include variables on the causal pathway for the correlate of interest,^23^ which can result in attenuated estimates.^24^ In addition, because the two models estimated for each correlate test only a single hypothesis, we did not adjust for multiple comparisons.^25^

Variables with missing values included institutionalization (n=5, 3%); number of students (n=1, 0.6%); teacher turnover (n=2, 1.2%); principal turnover (n=2, 1.2%); principal leadership (n=17, 10.4%); years HPI in school (n=10, 6.1%); families invited to participate (n=19, 11.7%); community groups invited to participate (n = 20, 12.3%); school board involved (n=17, 10.4%); program champion present (n=2, 1.2%); and modifications made to HPI (n=19, 11.7%). Missing values in institutionalization and potential correlates were accounted for using multiple imputation. Per von Hippel’s 2-step calculation to determine the number of imputation sets needed to produce replicable estimates of standard errors,^26^ we created 20 imputed datasets using predictive mean matching with 10 nearest neighbour comparators for continuous and ordinal variables,^27^ logistic regression for binary variables and negative binomial regression for number of students, which was overdispersed.^28^

## Results

***School key informants and school characteristics***

Of 171 elementary schools participating in Prome*SS*, 163 (95%) provided data on the index HPI and were retained for analysis. School key informants were principals (93%), vice-principals (4%) or teachers (3%) and had spent on average 3.4 years working in their school (SD=2.6, range= 1–10). Characteristics of participating elementary schools were similar to those of all eligible elementary schools in Quebec regarding school deprivation level (35% of participating vs. 38% of eligible schools served disadvantaged students),^19^ language of instruction (primarily French, 83%) and number of students.^13^ Fifty-six percent of participating schools were located in rural or small population centres (population≤29999). Finally, 42% and 22% of school key informants reported “some/a lot” of teacher and principal turnover in the past three years, respectively.

***Description of index HPIs***

Across the 163 participating schools, a total of 147 unique HPIs were selected by participants in the second part of the interview, some of which are described in previous work.^13^ These index HPIs addressed one or multiple health-related topics (e.g. physical activity and healthy eating,* personal safety and injury prevention, bullying,* aggressive behaviour, mental health, personal hygiene, puberty, addiction prevention, oral health* and tobacco prevention and education).^29^-^32^ Fifty-six percent of index HPIs (n=88) were institutionalized, and half had been in schools at least three years (interquartile range=2–6, range= 1–43). Among index HPIs related to mandated topics, all three that addressed oral health were institutionalized, as were 84% (36/43) addressing bullying, 45% (42/93) related to physical activity and 46% (32/70) related to healthy eating.

***School-related correlates of institutionalization***

Three aspects of health-promoting school culture (i.e. parent/community engagement in the school, school/teacher commitment to student health and the school’s physical environment) were positively associated with HPI institutionalization. None of the characteristics describing school structure or student demographics were associated with institutionalization (Table 3).

**Table 3:** Unadjusted and adjusted ORs and 95% CIs from logistic regression models for the association between school characteristics and institutionalization of school-based health-promoting interventions

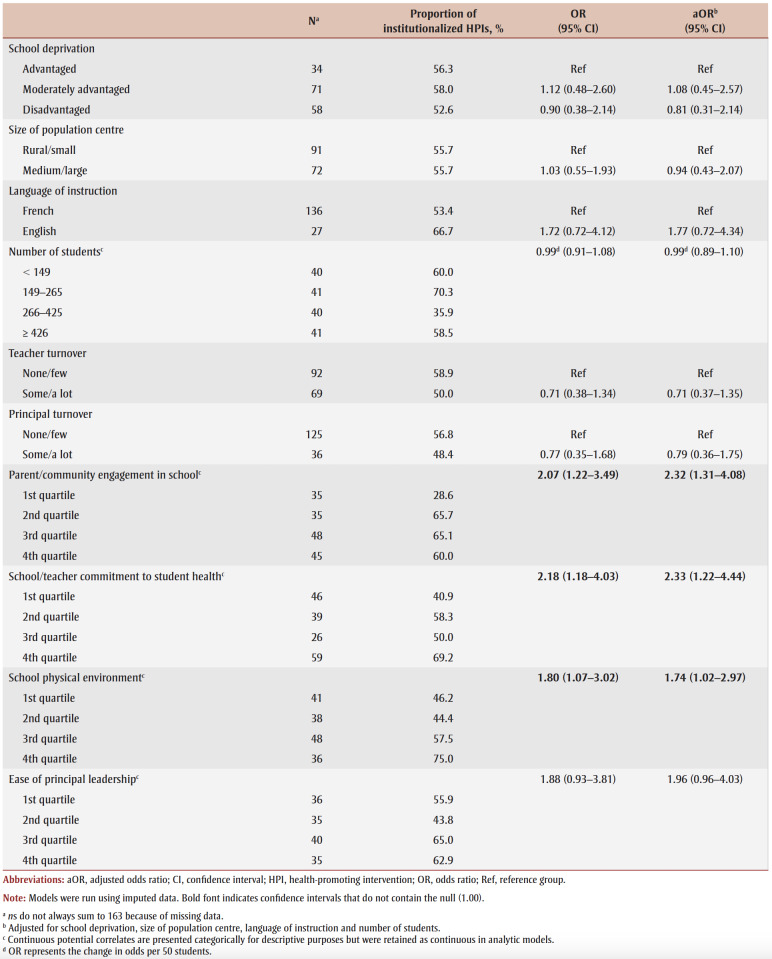

***HPI-related correlates of institutionalization***

Four HPI-related characteristics were positively associated with institutionalization of the index HPI (Table 4). HPIs that incorporated a greater number of core competencies or a larger number of teaching strategies, or both, were more likely to be institutionalized, as were HPIs that were modified during implementation or both prior to and during implementation. Additionally, the greater the perception that the HPI was successful, the higher the odds of institutionalization. Finally, HPIs in which families were invited to participate were less likely to be institutionalized than those that did not invite families. Adjusted odds ratios for variables with imputed values were within 0.09 of those obtained in sensitivity analyses with complete cases.

**Table 4:** Unadjusted and adjusted ORs and 95% CIs from logistic regression models for the association between intervention-related characteristics and institutionalization of school-based health-promoting interventions

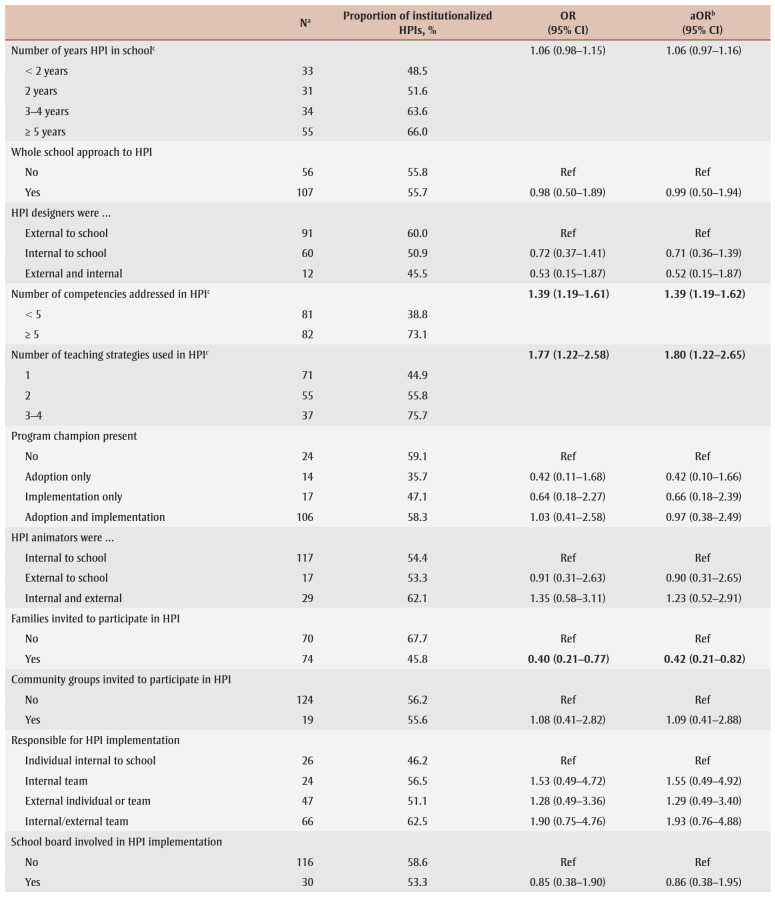 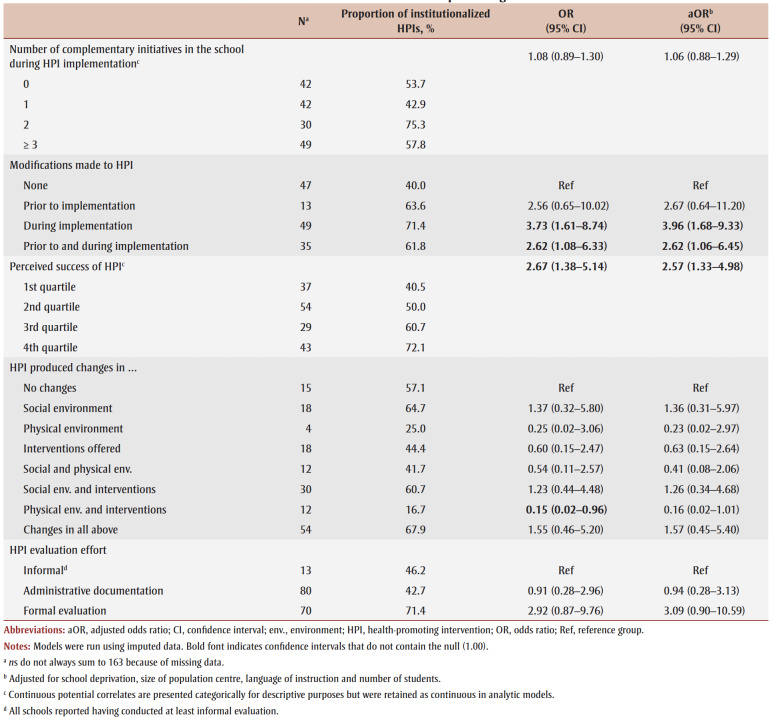

The effectiveness of an HPI can be determined only by formal evaluation of the extent to which the intervention met measurable benchmarks.^33^ In this study, institutionalization was unrelated to evaluation effort, yet, in an earlier study, the index HPIs were seen as highly successful.^16^ Perceived success was equivalent for HPIs evaluated informally (M [SD]=4.38 [0.58]), via documentation (4.19[0.52]) or via formal assessment (4.31[0.49]; *F*(2/160)=1.46, *p*=0.23).

*Mandated by the Quebec government for elementary schools.

## Discussion

In this study, we identified correlates of HPI institutionalization from among a comprehensive range of school- and HPI-related characteristics in elementary schools in Quebec, Canada. Benefits of school-based HPI institutionalization include HPI sustainability, consistency over time, accountability and scalability.^8^,^34^,^35^ Institutionalization is generally viewed as a positive step toward ensuring that HPIs can continue to benefit students after the initial implementation phase.^8^ In the current study, over half of the HPIs were institutionalized. The correlates of institutionalization identified herein are discussed below.

***School-related correlates of institutionalization***

School culture indicators associated with HPI institutionalization included more active involvement of parents and the community in the school, strong commitment to student health among school staff and a physical environment favourable to student health. Beliefs and norms shared within the school (i.e. school culture) are known to be driving forces of the operational processes and motivations that guide HPI implementation.^8^ Availability of health-promoting equipment and space could increase the likelihood of school staff choosing an HPI that aligns with the school context. Strong commitment to student health promotion among the school staff can positively influence perceptions of HPIs within the school community, especially in schools where staff believe in the relevance and importance of such interventions. Finally, our results support existing evidence that the role of school principals in guiding staff towards objectives, obtaining resources, distributing responsibilities and resolving conflicts is critical, with multiple studies highlighting the need for strong leadership to facilitate HPI implementation.^36^

Two systematic reviews recently investigated barriers and facilitators to sustainability of school-based HPIs targeting a variety of health themes.^7^,^34^ Although most interventions in these reviews were not completely sustained, Herlitz et al. identified four categories of factors associated with sustainment: school capacity to sustain HPIs, staff motivation and commitment to sustain HPIs, HPI adaptability and integration, and the wider policy context. Factors consistently related to HPI sustainability included leadership by school principals and administration, and commitment and confidence of school staff to promote health,^7^ which aligns with our findings.

Our findings also support previous work underscoring the importance of establishing a supportive environment in schools, either before introducing an HPI or as a target of intervention, as well as considering the school climate or culture for adapting an HPI.^21^,^37^ For long-term success, a comprehensive and collaborative approach is needed to address the complex public health challenges that many HPIs aim to tackle. The feasibility of improving school culture to increase access to and effectiveness of HPIs is demonstrated in Canada by the APPLE Schools initiative: an innovative, evidence-based HPI that fosters a supportive school culture to facilitate behaviour change (healthy eating, physical activity, mental well-being) in students.^38^-^40^

***HPI-related correlates of institutionalization***

Several characteristics of HPIs were associated with institutionalization. First, HPIs that integrated more core competencies and/or a wider range of teaching strategies were more likely to be institutionalized. Research suggests that multicomponent school-based HPIs are more likely than single-component interventions to meet benchmarks and be cost-effective and sustainable.^40^ Second, if an HPI was modified during or prior to implementation, it was more likely to be institutionalized. A “one size fits all” approach may overlook modifications and adaptations needed to render an HPI a good fit to the school.^41^ Each school has a unique environment, with its own student population, staff and culture, and what works in one school may not work in another. Modifying or adapting an HPI to the school culture and context is essential to increase its relevance, acceptability and effectiveness, integrate local resources and assets and ensure its institutionalization.^41^

Third, HPIs that included families were less likely to be institutionalized compared to those that did not involve families. This finding is intriguing because involving families and other external stakeholders has been recommended as important to HPI success.^42^-^44^ It is possible that there may be concerns around confidentiality and privacy or that excluding parents increases ease and efficiency of HPI implementation. Additional coordination and resources may be needed to ensure parental participation. However, parental involvement can be critical to HPI success, particularly in promoting healthy behaviours beyond the school environment.^42^-^44^ Thus, striking a balance between the advantages of institutionalization and the potential benefits of parental involvement is essential.

Finally, HPIs that were perceived as successful were more likely to be institutionalized. Perceived success can generate support and buy-in from key stakeholders, including school administrators, staff and parents, who may be more willing to allocate resources to the intervention over time.^8^

It is important to note that neither perceived success nor institutionalization of HPIs guarantees that they are (or remain) effective (i.e. meet established benchmarks), which can be assessed only through formal evaluation.^33^,^37^ Many school-based HPIs are not evaluated in practice for reasons related to lack of time and resources, and challenges in measuring health outcomes in the short- and long-term, and many HPIs are sustained despite being ineffective. In a survey of US public health practitioners from state and local health departments and related agencies, 36% to 42% reported that effective programs that should have continued were discontinued, and 25% to 29% reported that ineffective programs that should have been terminated were continued.^45^ Perceived success may not align with effectiveness when HPIs are not adequately evaluated. We suggest that the Ministry and school boards provide incentives to schools to evaluate the effectiveness of available HPIs in achieving measurable benchmarks, and that the evaluations be conducted on a regular basis to guard against decisions that are not evidence-based.

***Strengths and limitations***

Strengths of this study include the use of a structured interview to collect data, which allowed for expansion and clarification of respondents’ comments, and the exploration of aspects of a health-promoting school culture that have not been previously investigated in the context of HPI institutionalization. 

Limitations of this analysis include the convenience sample of schools, which could limit generalizability. However, the characteristics of Prome*SS* schools resembled those of all eligible elementary schools in Quebec. Responses from a single key informant within a school may not provide an accurate portrayal of the organizational perspective. However, data collection from multiple respondents within the same school was not feasible. In addition, the Prome*SS* questionnaire was sent to informants prior to the interview so that they could consult their staff to prepare. Our measure of institutionalization included a single item, and its validity and reliability are not established. Recall error could have resulted in misclassification bias in the observed associations. Our measures of health-promoting school culture are new and require further validation. Finally, participants might have been motivated to present the most desirable impression of their schools or chosen to discuss an HPI with which they were more familiar and, perhaps, which was more likely to have been institutionalized, which may have introduced bias.

## Conclusion

The work presented herein adds to a growing literature on factors associated with HPI institutionalization. These factors include indicators of health-promoting school culture (parent/community engagement with the school, school/teacher commitment to student health, school physical environment) as well as characteristics of the HPI (number of competencies addressed by the HPI, number of teaching strategies employed, modifications made to the HPI prior to or during implementation, perceived success of the HPI, not inviting families/community groups to participate in the HPI). Our findings therefore suggest that to optimize sustainability, characteristics of both the school context and the intervention itself must be considered in the design and implementation of HPIs.

## Acknowledgements

The PromeSS project was funded by the Quebec Ministry of Health and Social Services. Erin O’Loughlin holds a postdoctoral salary award from the Fonds de recherche du Qubec—Sant (FRQ-S). Katerina Maximova holds the Murphy Family Foundation Chair in Early Life Interventions. Jennifer O’Loughlin held a Canada Research Chair in the Early Determinants of Adult Chronic Disease from 2006 to 2021. Funders were not involved in the study design, data collection, analysis or interpretation, or the preparation of the manuscript for publication.

## Conflicts of interest

The authors have no competing interests.

## Authors’ contributions and statement

RW—supervision, methodology, formal analysis, writing—original draft, writing—review & editing. 

EOL—conceptualization, methodology, writing—original draft, writing—review & editing.

KM—supervision, conceptualization, writing—review & editing. 

JK—conceptualization, writing—original draft, writing—review & editing. 

TR—resources, conceptualization, writing—original draft, writing—review & editing. 

JOL—conceptualization, resources, methodology, writing—original draft, writing—review & editing, supervision, project administration, funding acquisition. 

All authors approved the final manuscript as submitted and agree to be accountable for all aspects of the work.

The content and views expressed in this article are those of the authors and do not necessarily reflect those of the Government of Canada.
